# Disparities in COVID-19 Testing and Infection Among Beneficiaries in the Military Health System During the First Year of the Pandemic

**DOI:** 10.1007/s40615-025-02315-x

**Published:** 2025-02-21

**Authors:** Elta Liang, Kevin Chuang, Kevin K. Chung, Christian L. Coles, Tracey Pérez Koehlmoos

**Affiliations:** 1 Uniformed Services University of the Health Sciences, Bethesda, MD USA; 2 The Henry M. Jackson Foundation for the Advancement of Military Medicine, Inc, 6720A Rockledge Drive, Suite 205, Bethesda, MD 20817 USA; 3 SeaStar Medical, Denver, CO USA

**Keywords:** COVID-19 testing, COVID-19 infections, COVID-19 pandemic, Healthcare disparities, Military health services, Universal coverage

## Abstract

**Background:**

Although disparities in access to COVID-19 testing and infection rates were identified in civilian literature, it is unclear whether the universally-insured U.S. Military Health System (MHS) experienced similar inequities. We examined whether there were disparities by race, sex, and rank within the MHS’ direct care sector during the early pandemic period.

**Methods:**

Retrospective study of adult TRICARE beneficiaries from March 1, 2020 to February 28, 2021. Likelihood of COVID-19 testing and infection, among eligible beneficiaries, for each exposure variable was assessed using logistic regression.

**Results:**

697,769 beneficiaries received COVID-19 testing during the study period with 56,037 testing positive. Women were more likely to be tested than men (OR: 1.23, 95% CI: 1.21–1.24), but less likely to test positive (OR: 0.87, 95% CI: 0.85–0.89). Compared to White beneficiaries, Black and Asian/Pacific Islander beneficiaries were more likely to be tested (OR: 1.07, 95% CI: 1.07–1.08; OR: 1.23, 95% CI: 1.21–1.24). Black beneficiaries were more likely to test positive (OR: 1.10, 95% CI: 1.07–1.13). Junior Enlisted members were less likely, while Junior Officers were more likely to be tested than Senior Enlisted members (OR: 0.73, 95% CI: 0.73–0.74; OR: 1.20, 95% CI: 1.18–1.21). Junior and Senior officers were less likely to test positive (OR: 0.92, 95% CI: 0.89–0.95; OR: 0.70, 95% CI: 0.67–0.74).

**Conclusion:**

Despite universal healthcare coverage, disparities in COVID-19 testing and infection rates by race, sex, and sponsor rank were identified within the MHS. Further research of underlying factors of observed disparities and targeted outreach are necessary for equitable care.

**Supplementary Information:**

The online version contains supplementary material available at 10.1007/s40615-025-02315-x.

## Background

In the United States (U.S.), SARS-CoV-2 accounted for over 500,000 deaths and approximately 3,000,000 hospitalizations during the first year of the COVID-19 pandemic [1, 2]. Both early diagnosis and prompt treatment were key to reducing severe morbidity and mortality due to COVID-19.

To enable case isolation and rapid treatment, the U.S. prioritized the development of diagnostic tests that could be distributed to all communities. By February 2020, the Food and Drug Administration (FDA) granted emergency use authorization to a first set of RT-PCR, or reverse transcription-polymerase chain reaction, diagnostic panels for COVID-19 testing [3]. Despite efforts to make COVID-19 testing universal and equitable, evidence quickly emerged suggesting that access to testing and test positivity rates differed by an individual’s socioeconomic status, race, and sex [4–10]. A national study of COVID-19 testing in 2020 reported that those without health insurance were significantly less likely to be tested but more likely to have positive test results than those who were insured [5]. Studies conducted across multiple regions of the U.S. found varying results in terms of testing rates by race. While some cohort studies found that minority groups, in particular Black adults, had lower testing rates, others found higher testing rates in non-White adults [6–8]. Despite this discrepancy, test positivity was consistently found to be higher among non-White individuals [6–8]. Across studies, females were more likely to be tested than males, yet infection rates were higher in males [8–10].

Similar to the civilian healthcare system, the U.S. Military Health System (MHS) was responsible for providing equitable access to testing for its beneficiaries during the COVID-19 pandemic. However, to date no studies have examined whether the same COVID-19 disparities identified in civilian healthcare were also present among all MHS beneficiaries. The MHS differs from the standard U.S. healthcare system in that it provides guaranteed access to care for a universally insured population [11]. Through TRICARE insurance, beneficiaries can receive either direct care at a military treatment facility (MTF) at no cost or private sector care from TRICARE-affiliated civilian facilities at minimal cost to the patient [12]. This system has been shown to mitigate barriers in access to health services such as screenings [11, 12].

TRICARE’s universal insurance model presents a unique opportunity to analyze disparities in healthcare for a large, nationally representative population [13]. Identifying and addressing disparities is a matter of ethical responsibility, as it is imperative to ensure that all beneficiaries receive essential healthcare and protection from disease. The importance is further justified by studies from the United Kingdom where healthcare coverage is universal, which found clear disparities in access to testing and infection rates [14, 15]. In this context, we sought to examine whether there were disparities in COVID-19 testing and infection rates among TRICARE beneficiaries within the direct care sector during the first year of the COVID-19 pandemic. Given the MHS’s universal healthcare coverage, we hypothesized that there would be minimal disparities in COVID-19 testing and infection rates by race, sex, and sponsor rank.

## Methods

### Study Design, Population, and Data Source

This retrospective study utilized claims data from the Military Health System Data Repository (MDR) to identify adult TRICARE Prime and Plus beneficiaries ages 18–64 during the first year of the pandemic (March 1, 2020 – February 28, 2021). Claims captured in the MDR include both inpatient and outpatient encounters and services. Individuals were eligible if they had a medical encounter (inpatient or outpatient) in one of more than 400 military treatment facilities in the direct care system within the study period. Medicare eligible beneficiaries (i.e. patients ages 65 and older) were excluded from the study due to a loss of visibility in the MDR as any claims paid for by Medicare or other health insurance are not recorded in the data.

We used laboratory data from the MDR along with Current Procedural Terminology/Healthcare Common Procedure Coding System (CPT/HCPCS) codes to identify all PCR-based COVID-19 tests. All CPT/HCPCS codes can be found in Online Resource 1. The identified COVID-19 tests were then matched to their corresponding results. Only those in direct care had their COVID-19 tests matched due to laboratory results being unavailable for private care patients. If patients received multiple tests, then the first record of testing was retained for analysis. Demographic information including sex, categorical age, self-reported race (White, Black, Asian/Pacific Island, American Indian/Alaska Native), beneficiary status (active duty, dependent, retiree), socioeconomic status, and associated branch of service (Army, Air Force, Navy, Marine Corps) were obtained from the beneficiary’s Defense Enrollment Eligibility Reporting System (DEERS) record and utilized in analysis. Sponsor rank (Junior Enlisted, Senior Enlisted, Junior Officer, Senior Officer, Warrant Officer) was utilized as a proxy for the socioeconomic status of TRICARE beneficiaries with enlisted rank being a correlate of lower socioeconomic status as demonstrated in previous studies of this population [16–18]. Due to their small size, beneficiaries associated with the US Space Force were grouped together with those in the US Air Force. Ethnicity was not retained in this analysis due to high rates of missing data.

### Statistical Analysis

Study analyses included descriptive statistics on patient demographics and frequency of COVID-19 testing overall and by sex, race and sponsor rank. We employed simple and multivariable logistic regression models to obtain unadjusted and adjusted odds ratios for patient likelihood of receiving a COVID-19 test among all eligible beneficiaries and the likelihood of testing positive among patients who received testing during the study period. The multivariable models included sex, race, and sponsor rank as independent predictors for receipt of COVID-19 testing and test positivity. Categorical age and beneficiary status were included in our multivariable models as potential confounders as they frequently appear in research conducted on MHS beneficiaries as covariates of interest and potential confounders [11].

Statistical significance was determined, a priori, as odds ratio (OR) and 95% confidence interval (CI) exclusive of 1.0 and α = 0.05. This study was reviewed and found exempt by the Institutional Review Board at the Uniformed Services University of the Health Sciences. All statistical analyses were conducted using SAS 9.4 (SAS Institute Inc). We followed the Strengthening the Reporting of Observational Studies in Epidemiology (STROBE) reporting requirements.

## Results

**Fig. 1:**
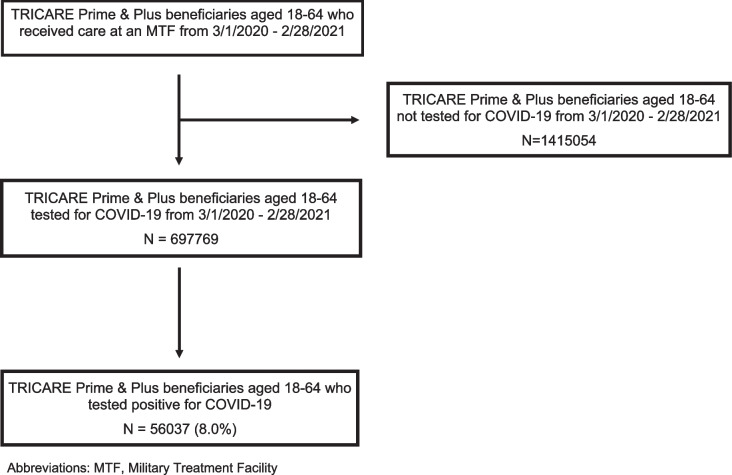
CONSORT diagram of eligible beneficiaries

During the study period (March 1, 2020 – February 28, 2021) we identified 2,112,823 eligible individuals with medical encounters in the direct care sector. Among those beneficiaries, 697,769 (33%) received testing for COVID-19 (Fig. 1). Of those receiving COVID-19 tests, the majority were male (70%), of White race (60%), ages 18–34 (70%), active duty (78%), and in an enlisted rank (80%) (Table 1). Additionally, 56% of the patients receiving COVID-19 tests during the study period received multiple tests (data not shown). Of those who received a COVID-19 test, 13,143 (2%) had canceled tests or missing results, while 56,037 (8%) tested positive.

**Table 1:** Demographics of eligible beneficiaries by receipt of COVID-19 testing

Variable	Total receiving care *N* (% of total)	Did not receive testing *n* (% of total)	Received testing ^a^		
			Total tests ordered *n* (% of total)	Positive test *n* (% of total)	Negative test *n* (% of total)
Total	2112823	1415054	697769	56037	628589
Age, y					
18–24	611733 (29.0)	366635 (25.9)	245098 (35.1)	19072 (34.0)	221277 (35.2)
25–34	653251 (30.9)	405048 (28.6)	248203 (35.6)	18468 (33.0)	224987 (35.8)
35–44	378397 (17.9)	257828 (18.2)	120569 (17.3)	9165 (16.4)	109232 (17.4)
45–54	229930 (10.9)	181575 (12.8)	48355 (6.9)	5107 (9.1)	42492 (6.8)
55–64	239512 (11.3)	203968 (14.4)	35544 (5.1)	4225 (7.5)	30601 (4.9)
Sex					
Female	808828 (38.3)	597420 (42.2)	211408 (30.3)	18394 (32.8)	189277 (30.1)
Male	1303995 (61.7)	817634 (57.8)	486361 (69.7)	37643 (67.2)	439312 (69.9)
Race ^b^					
White	1235943 (58.5)	812873 (57.4)	423070 (60.6)	32664 (58.3)	381711 (60.7)
Black	334465 (15.8)	220305 (15.6)	114160 (16.4)	9919 (17.7)	102383 (16.3)
Asian/Pacific Islander	127896 (6.1)	77092 (5.5)	50804 (7.3)	3529 (6.3)	46178 (7.3)
American Indian/Alaska Native	15022 (0.7)	8594 (0.6)	6428 (0.9)	515 (0.9)	5756 (0.9)
Other	234315 (11.1)	180060 (12.7)	54255 (7.8)	4778 (8.5)	48706 (7.8)
Missing	165182 (7.8)	116130 (8.2)	49052 (7.0)	4632 (8.3)	43855 (7.0)
Beneficiary status					
Active duty	1237356 (58.6)	692430 (48.9)	544926 (78.1)	38278 (68.3)	496182 (78.9)
Dependent	629677 (29.8)	515606 (36.4)	114071 (16.4)	12696 (22.7)	99423 (15.8)
Retiree	245072 (11.6)	206468 (14.6)	38604 (5.5)	5048 (9.0)	32834 (5.2)
Other	718 (0.0)	550 (0.0)	168 (0.0)	15 (0.0)	150 (0.0)
Branch					
Army	829780 (39.3)	561324 (39.7)	268456 (38.5)	20679 (36.9)	245246 (39.0)
Air Force/Space Force	553966 (26.2)	398620 (28.2)	155346 (22.3)	13142 (23.5)	137853 (21.9)
Navy	473687 (22.4)	284839 (20.1)	188848 (27.1)	14024 (25.0)	171235 (27.2)
Marine Corps	223729 (10.6)	146207 (10.3)	77522 (11.1)	7466 (13.3)	67515 (10.7)
Other	31661 (1.5)	24064 (1.7)	7597 (1.1)	726 (1.3)	6740 (1.1)
Rank					
Junior Enlisted	522857 (24.8)	291919 (20.6)	230938 (33.1)	18024 (32.2)	208204 (33.1)
Senior Enlisted	788037 (37.3)	462900 (32.7)	325137 (46.6)	28302 (50.5)	290801 (46.3)
Junior Officer	166480 (7.9)	81254 (5.7)	85226 (12.2)	6256 (11.2)	77333 (12.3)
Senior Officer	112865 (5.3)	83679 (5.9)	29186 (4.2)	2094 (3.7)	26622 (4.2)
Warrant Officer	31358 (1.5)	17805 (1.3)	13553 (1.9)	1151 (2.0)	12252 (2.0)
Other	491226 (23.2)	477497 (33.7)	13729 (2.0)	210 (0.4)	13377 (2.1)

^a^ Positive and negative results do not total the number of tests ordered due to canceled tests and missing results (*n* = 13143 [2%])

^b^ In the Military Health System Data Repository, race does not include ethnicity; thus, categories reported are race only. Race is self-reported and Military Health System beneficiaries could select the Other race value to identify as multiracial; however, details regarding race combinations are not available or reported

Our adjusted analysis of all eligible patients showed that female beneficiaries had increased odds of receiving COVID-19 testing (OR: 1.23, 95% CI: 1.21–1.24) compared to male beneficiaries (Table 2). When assessing patient likelihood of receiving a COVID-19 test by race, Black beneficiaries (OR: 1.07, 95% CI: 1.07–1.08), Asian/Pacific Islander beneficiaries (OR: 1.23, 95% CI: 1.21–1.24), and American Indian/Alaska Native beneficiaries (OR: 1.22, 95% CI: 1.17–1.26) were more likely to receive COVID-19 testing compared to White beneficiaries. For sponsor rank, only Junior Officers (OR: 1.20, 95% CI: 1.18–1.21) and Warrant Officers (OR: 1.18, 95% CI: 1.15–1.21) had an increase in their odds of receiving a COVID-19 test compared to Senior Enlisted.

**Table 2:** Logistic regression results for receipt of COVID-19 testing ^a^

Variable	Unadjusted OR (95% CI) *N* = 2112823	Adjusted OR (95% CI) *N* = 2112823
Sex		
Female	0.59 (0.59–0.60) ^b^	1.23 (1.21–1.24) ^b^
Male	1 [Reference]	1 [Reference]
Race ^c^		
White	1 [Reference]	1 [Reference]
Black	1.00 (0.99–1.00)	1.07 (1.07–1.08) ^b^
Asian/Pacific Islander	1.27 (1.25–1.28) ^b^	1.23 (1.21–1.24) ^b^
American Indian/Alaska Native	1.44 (1.39–1.49) ^b^	1.22 (1.17–1.26) ^b^
Other	0.58 (0.57–0.59) ^b^	0.78 (0.77–0.79) ^b^
Missing	0.81 (0.80–0.82) ^b^	1.72 (1.70–1.75) ^b^
Rank		
Junior Enlisted	1.13 (1.12–1.13) ^b^	0.73 (0.73–0.74) ^b^
Senior Enlisted	1 [Reference]	1 [Reference]
Junior Officer	1.49 (1.48–1.51) ^b^	1.20 (1.18–1.21) ^b^
Senior Officer	0.50 (0.49–0.50) ^b^	0.56 (0.55–0.56) ^b^
Warrant Officer	1.08 (1.06–1.11) ^b^	1.18 (1.15–1.21) ^b^
Other	0.04 (0.04–0.04) ^b^	0.02 (0.01–0.02) ^b^

Abbreviations: OR, odds ratio; CI, confidence interval

^a^ Multivariable logistic regression models were adjusted by categorical age and beneficiary status

^b^ Indicates statistical significance with *p* < 0.05

^c^ Race is self-reported and Military Health System beneficiaries could select the Other race value to identify as multiracial; however, details regarding race combinations are not available or reported

In our secondary analyses, we assessed patient likelihood of testing positive for COVID-19 among those who received testing (excluding those with canceled tests or missing results). Female beneficiaries (OR: 0.87, 95% CI: 0.85–0.89) were less likely to test positive compared to male beneficiaries (Table 3). In comparison to those of White race, Black beneficiaries (OR: 1.10, 95% CI: 1.07–1.13) had a greater likelihood of testing positive, while Asian/Pacific Islander beneficiaries (OR: 0.94, 95% CI: 0.91–0.98) had a lower likelihood of testing positive for COVID-19. When compared to Senior Enlisted, other sponsor ranks were less likely to test positive for COVID-19.

**Table 3:** Logistic regression results for patients testing positive for COVID-19 ^a^

Variable	Unadjusted OR (95% CI) *N* = 684626	Adjusted OR (95% CI) *N* = 684626
Sex		
Female	1.13 (1.11–1.16) ^b^	0.87 (0.85–0.89) ^b^
Male	1 [Reference]	1 [Reference]
Race ^c^		
White	1 [Reference]	1 [Reference]
Black	1.13 (1.11–1.16) ^b^	1.10 (1.07–1.13) ^b^
Asian/Pacific Islander	0.89 (0.86–0.93) ^b^	0.94 (0.91–0.98) ^b^
American Indian/Alaska Native	1.05 (0.96–1.15)	1.05 (0.96–1.15)
Other	1.15 (1.11–1.18) ^b^	0.98 (0.95–1.02)
Missing	1.23 (1.20–1.28) ^b^	1.00 (0.96–1.03)
Rank		
Junior Enlisted	0.89 (0.87–0.91) ^b^	0.98 (0.95–1.00)
Senior Enlisted	1 [Reference]	1 [Reference]
Junior Officer	0.83 (0.81–0.86) ^b^	0.92 (0.89–0.95) ^b^
Senior Officer	0.81 (0.77–0.85) ^b^	0.70 (0.67–0.74) ^b^
Warrant Officer	0.97 (0.91–1.03) ^b^	0.92 (0.87–0.98) ^b^
Other	0.16 (0.14–0.19) ^b^	0.18 (0.16–0.21) ^b^

Abbreviations: OR, odds ratio; CI, confidence interval

^a^ Multivariable logistic regression models were adjusted by categorical age and beneficiary status

^b^ Indicates statistical significance with *p* < 0.05

^c^ Race is self-reported and Military Health System beneficiaries could select the Other race value to identify as multiracial; however, details regarding race combinations are not available or reported

## Discussion

In this investigation of TRICARE beneficiaries who received care at a Military Treatment Facility, we identified 697,769 individuals who were tested for COVID-19. We compared rates of COVID-19 testing and infection by various categories among beneficiaries to assess for potential disparities. Our study population was demographically representative of the MHS population as a whole during the study period [13]. In line with studies from the United Kingdom which has universal healthcare coverage, our analysis revealed disparities in testing utilization and disproportionate rates of infections in the MHS across various sociodemographic categories [14, 15]. Specifically, disparities by sex, race, and sponsor rank were observed within the MHS despite the provision of universal healthcare coverage.

With regards to race, our findings showed that beneficiaries from minority backgrounds (Black, Asian/Pacific Islander, and American Indian/Alaska Native) were more likely to receive testing than White beneficiaries. However, there were noticeable disparities in infection rates, with Black beneficiaries being more likely and Asian/Pacific Islander beneficiaries being less likely to test positive compared to White beneficiaries. Our findings aligned partially with research conducted within the military. A study of racial disparities in COVID-19 infections among active duty service members during 2020 found that while Black and White service members had relatively similar testing rates, Black service members had a higher risk of testing positive than White service members [19]. Two studies conducted within the civilian population, one analyzing health system data from California and the other assessing data from the US Department of Veterans Affairs, both found that testing and infection rates were higher among racial minorities, in particular Black, Hispanic, and Asian individuals, compared to White individuals [6, 20].

The heterogeneity between our findings and other studies suggest that there are other factors, such as working conditions, health literacy, and trust in the healthcare system that may be contributing to differences in COVID-19 testing and infection rates among various racial groups [21, 22]. The one consistent observation between our findings and previously described studies is that Black beneficiaries were more likely to test positive for COVID-19. This suggests that universal healthcare access may not be sufficient to fully address disparities in COVID-19 infection rates among Black individuals, and that special attention is needed to improve care among this group. This is especially important in light of the numerous studies demonstrating that Black individuals with COVID-19 were at increased risk for hospitalization and severe outcomes [23–25].

Similar to many studies conducted within the civilian population, our study found that while female beneficiaries were more likely to be tested for COVID-19, they were less likely to test positive than male beneficiaries [9, 10, 20]. Several studies have proposed potential reasons for the observed sex-based disparities in COVID-19 testing and infection. In a national study of perceptions towards COVID-19, researchers found that women had a greater tendency to perceive COVID-19 as a major population health risk and to report higher levels of fear surrounding coronavirus in comparison to men [26]. Additionally, multiple studies have reported that women were more likely than men to follow preventive health practices such handwashing, mask wearing, and social distancing during the COVID-19 pandemic [27–29]. Similarly, studies have found that men are less likely to engage in health seeking behaviors such as screenings and are more likely to participate in high-risk behaviors, both of which may impact their approach to combating COVID-19 [28, 29]. Although we can only speculate, the observed disparities in COVID-19 testing and infections between male and female beneficiaries in our study likely result from a complex interplay of societal, occupational, and behavioral factors.

Notably, our analysis showed that with the exception of Senior Officers, Officer ranks were more likely to be tested for COVID-19 than enlisted ranks. Despite being more likely to receive testing, Officer ranks were less likely to test positive than Senior Enlisted service members. One plausible explanation for the heterogeneity in testing rates by rank is the prioritization of testing for particular groups in the military due to limited resources. In early 2020, the FDA and other government agencies cited national shortages in testing resources that were attributed to insufficient domestic production and overwhelming global demand [30]. Accordingly, the Department of Defense (DOD) developed a tiered framework for prioritizing testing, which was dependent on service member’s missions [30]. Further examination of the relationship between the military ranks of our study population and the DOD’s tiered testing framework may uncover reasons for the observed differences. With regards to test positivity, our findings correspond with other literature regarding socioeconomic disparities in COVID-19 infections during the early pandemic. A systematic review of studies on COVID-19 disparities between 2019 and 2021 found higher infection risk in those with low socioeconomic status, as indicated by measures such as low income and lower educational attainment [31]. Similarly, our findings revealed that Officer rank, a military correlate of higher socioeconomic status, was associated with a lower likelihood of testing positive for COVID-19.

We acknowledge that there are several limitations to this study. Due to COVID-19 lab results being unavailable in the MDR for private sector care patients, we were only able to present results for beneficiaries who received care in the direct care system. Given that this was a retrospective study of healthcare claims data, we were unable to gather additional information in the event of missing data. We realize that 56% of the study sample received multiple tests and understand that patients who have previously tested may be more likely to be tested again. This did not affect the analysis as only the first record of testing was retained and used in our analysis. We recognize that 7.8% of the race data was missing for all medical encounters during the study period, and 7% of those tested for COVID-19 had missing race data. However, it is important to note that race is self-reported in the MHS and is not a requirement for medical care. We were cognizant of the need to better understand the associations between race and COVID-19 related care. Additionally, we cannot account for inconsistencies in coding practices between providers and recognize this as a possible source of error in the analysis. Nonetheless, we believe that our data is highly representative of PCR-based COVID-19 testing within the direct care sector of the MHS during the study period.

## Conclusions

Despite the MHS providing universal healthcare coverage to its beneficiaries, our study found significant disparities in COVID-19 testing and infections rates by race, sex, and sponsor rank. The identification of such disparities within a population that has universal insurance coverage may raise significant ethical concerns. Further research into the underlying factors that may be contributing to disproportionate rates of testing utilization and likelihood of infection is necessary to ensure that all MHS beneficiaries are taking preventative measures and receiving necessary healthcare services. It is imperative to design targeted outreach plans that take into consideration factors such as health literacy, perceptions of the healthcare system, socio-behavioral influences, and occupational demands among MHS beneficiaries. National efforts to better prepare for potential disease outbreaks and ensure that enough testing resources are available to prioritize all populations may help to protect vulnerable groups and mitigate disparities. Taken together, these measures may strengthen the MHS’s ability to deliver equitable care to all of its beneficiaries.

## Supplementary Information

Below is the link to the electronic supplementary material.

ESM 1 (PDF 77.4 KB)  

## Data Availability

The data that support the findings of this study are available from the United States Defense Health Agency (DHA). Restrictions apply to the accessibility of these data, which were used under Federal Data User Agreements for the current study, and so are not publicly available.
