# Supplementary material for: Disparities in COVID-19 Testing and Infection Among Beneficiaries in the Military Health System During the First Year of the Pandemic
Source: J Racial Ethn Health Disparities. 2025 Feb 21;13(2):1065–72. doi: 10.1007/s40615-025-02315-x (PMC12966207; doi:10.1007/s40615-025-02315-x)
Supplement: Supplementary file 1 — (PDF 77.4 KB) [file 40615_2025_2315_MOESM1_ESM.pdf]

Online Resource 1. CPT/HCPCS Codes for PCR-based COVID-19 Tests

CPT/HCPCS	Description
87635	Infectious agent detection by nucleic acid (DNA or RNA); severe acute respiratory syndrome coronavirus 2 (SARS-CoV-2) (Coronavirus disease [COVID-19]), amplified probe technique
87636	Infectious agent detection by nucleic acid (DNA or RNA); severe acute respiratory syndrome coronavirus 2 (SARS-CoV-2) (Coronavirus disease [COVID-19]) and influenza virus types A and B, multiplex amplified probe technique
87637	Infectious agent detection by nucleic acid (DNA or RNA); severe acute respiratory syndrome coronavirus 2 (SARS-CoV-2) (Coronavirus disease [COVID-19]), influenza virus types A and B, and respiratory syncytial virus, multiplex amplified probe technique
0202U	Infectious disease (bacterial or viral respiratory tract infection), pathogen specific nucleic acid (DNA or RNA), 22 targets including severe acute respiratory syndrome coronavirus 2 (SARS-CoV-2), qualitative RT-PCR, nasopharyngeal swab, each pathogen reported as detected or not detected

Abbreviations: CPT/HCPCS, Current Procedural Terminology/Healthcare Common Procedure Coding System; DNA, Deoxyribonucleic acid; RNA, Ribonucleic acid; RT-PCR, Reverse Transcription Polymerase Chain Reaction
